# Digital PCR Validation for Characterization of Quantitative Reference Material of *Escherichia coli* O157*:H7* Genomic DNA

**DOI:** 10.3390/mps7060094

**Published:** 2024-11-15

**Authors:** Claudia Patricia Tere-Peña, Martha Nancy Calderon-Ozuna, John Emerson Leguizamón Guerrero

**Affiliations:** 1 Department of Chemistry, Faculty of Science, Universidad Nacional de Colombia, Bogotá 111321, Colombia; mncalderono@unal.edu.co (M.N.C.-O.); jeleguizamon@inm.gov.co (J.E.L.G.); 2 Sub-Directorate of Chemical Metrology and Biology, Instituto Nacional de Metrología de Colombia, Bogotá 111321, Colombia

**Keywords:** *E. coli* O157:H7, reference material, method validation, digital PCR

## Abstract

*Escherichia coli O157:H7*, a Shiga-toxin-producing *E. coli* (STEC), is an important pathogen related to foodborne disease that is responsible for a growing number of outbreaks worldwide and has been detected in processed meats, dairy, and fresh vegetables. Although culturing is the gold standard method for detection of this bacterium, molecular methods based on nucleic acid amplification techniques such as PCR are becoming more common because of their rapidity, sensitivity, and specificity. However, to ensure reliable results among the several alternative PCR protocols (e.g., commercial kits and reference methods), different measurement assurance tools, including validated methods, reference materials, and proficiency tests, among others, are required. Herein, we present a digital PCR method validation for *E. coli O157:H7* detection and quantification using seven specific gene sequences; this method quantified nucleic acids from different *E. coli* serotypes, with a detection range of 6.6 to 7900 copies/µL and a repeatability standard deviation over the concentration range of 1% to 13.6%. The relative standard uncertainty was 3.5–14.6%, and the detection limit was 0.27 copies/µL. Subsequently, two batches of a candidate reference material based on *E. coli* O157:H7 genomic DNA were then produced and characterized for evaluation of copy number concentration with the validated ddPCR method, with assigned values of 164,770 ± 9251 and 172 ± 9 copies/μL. Thus, this study demonstrated the development of a validated method and reference material for dPCR and qPCR detection of *E. coli* O157:H7, a key STEC responsible for food poisoning.

## 1. Introduction

Enterohemorrhagic *Escherichia coli*, a subgroup of Shiga-toxin-producing *E. coli* (STEC), causes intestinal and renal diseases, including hemorrhagic colitis and hemolytic uremic syndrome, and even death. The representative strain is *E. coli* O157:H7, which was identified in an outbreak of bloody diarrhea in 1983 [1]. This bacterium has subsequently been detected in foods such as processed meats, dairy, and fresh vegetables and is responsible for an increasing number of outbreaks globally [2]. Detection of *E. coli* O157:H7 and other non-O157 STEC is playing a key role in diagnostics, environmental protection, and food safety.

Microbiological culture is the gold standard method for *E. coli* O157:H7 detection [3]; however, this method is time-consuming as samples often contain low target cell numbers and high levels of background flora and natural inhibitors that interfere with isolation. To address these issues, several methods for rapid identification based on nucleic acid amplification techniques such as PCR have been developed [4]. PCR has become a powerful diagnostic tool for detection of pathogenic microorganisms in food samples because of advantages such as specificity, sensitivity, and high throughput [5].

PCR detection of STEC is based on the amplification of virulence gene sequences conserved in several serotypes of this group. The most common genes include (i) *stx1* and *stx2* that encode Shiga-like toxins that have N-glycosylase RNA activity and thus inhibit protein synthesis [6], (ii) *rfbE* that encodes an enzyme associated with membrane lipopolysaccharide biosynthesis in outer membrane biogenesis and is related to O-antigens [6], (iii) *eaeA* that encodes an adhesion protein involved in the invasion of *E. coli* into enterocytes and is an integral component of the membrane [7], and (iv) *Z3276*, a specific genetic marker of STEC O157:H7 involved in biofilm formation. Other representative genes for genus detection are *uidA*, which is used to identify *E. coli* serotypes, as this encodes a β-glucuronidase, although this may be present in *Shigella* strains, [8] and *lacY*, which encodes a beta-galactoside transporter that is used to discriminate between *E. coli* and *Shigella* strains [9,10].

PCR analysis methods can be qualitative, semi-quantitative, or quantitative, depending on the analysis purpose, and many of them rely on using standard references to prepare calibration curves as real-time or quantitative PCR (qPCR). Recently, digital PCR (dPCR) has evolved as the latest PCR generation system for absolute quantification of nucleic acids [11,12]. It is based on a sample partitioning into many small containers, where each partition can contain a discrete number of biological entities and, after an end point reaction, a quantification (in copies/µL) based on the fraction of positive partitions is performed [13]. Depending on the partitioning approach, droplet digital PCR (ddPCR) and chip digital PCR (cdPCR) formats are available.

One prerequisite to facilitate the development of PCR methods is the availability of DNA standards [14]. Manufacturers usually develop their own standards for use as internal or positive controls. This practice gives rise to systematic error and consequently produces less comparable results between laboratories [15]. In this situation, DNA reference materials (RMs) could help to reduce the within and among variability in laboratory quantitation [16], allowing a guarantee of the reliability and traceability of PCR results. The dPCR could be considered as a potential primary method because this can quantify DNA without a standard or internal control [17] and can be used to characterize DNA in the production of RMs [18,19].

Thus, the purpose of this study was (i) to describe a droplet digital PCR (ddPCR) method validation for *E. coli* O157:H7 quantification using specific gene sequences and (ii) to describe the use of this method to characterize a genomic DNA (gDNA) RM. For the validation, the performance characteristics evaluated were working interval, limit of detection (LOD), limit of quantification (LOQ), selectivity, precision, and uncertainty [20]. Subsequently, the RM candidate was prepared at two concentration levels in solution. We performed a homogeneity and stability study to assign a copy number concentration value with its uncertainty. This work aimed to contribute to standardization of *E. coli* O157:H7 detection by PCR assays and improve interlaboratory comparisons toward the strengthening of monitoring processes in agriculture and environmental industries.

## 2. Materials and Methods

### 2.1. Reagents and Instruments

The IRMM 449^®^ gDNA of *E. coli O157* strain EDL933, certified in identity, was used as a calibrant for PCR amplification [21]. Lyophilized DNA was solubilized in 1X TE buffer (10 mM Tris, 1 mM EDTA, pH 8.0) at a nominal concentration of 13.0 ng/µL (approximately 2.14 × 10^6^ genome copies/µL). Six 1:10 serial dilutions were prepared gravimetrically. ERM-AD623-certified RM was used as a quality control to evaluate the performance of the ddPCR method [20]. 

Primers and probes were synthesized by Integrated DNA Technologies (Coralville, IO, USA) and were solubilized in 1X TE buffer, diluted to 10 mM, and stored at −30 °C. Droplet digital PCR (ddPCR) was performed on the QX200 digital PCR system (Bio-Rad for Hercules, CA, USA, cat. 186-40031). The CFX96 touch deep well thermal cycler (Bio-Rad cat. 3600037) was used for both qPCR and ddPCR. Large-scale DNA extraction was performed using a Sorvall Lynx 4000 centrifuge (Thermo Fisher Scientific, Waltham, MA, USA) with Bioflex HC and TH13 rotors (Thermo Fisher Scientific, Waltham, MA, USA), whereas for small-scale DNA extraction, a Heraus™ Megafuge™ 16R centrifuge (Thermo Fisher Scientific, Waltham, MA, USA) with Microliter 30 × 2 rotor was used.

All KWIK-STIK™ of bacterial strains used in selectivity studies of PCR, were purchased from Microbiologics (Saint Cloud, MN, USA), were preserved at 4 °C until use. *E. coli* strain O157:H7 derived from ATCC 35150 was used for RM production.

### 2.2. ddPCR Method Validation

#### 2.2.1. Primer and Probe Design

Seven single-copy, highly conserved genes frequently used for the detection of *E. coli* spp. and *E. coli* O157:H7 by PCR were selected based on currently available literature and used as target genes: *uidA, lacY, eaeA, rfbE, stx1, stx2*, and *Z3276*. Specific primers and probes were selected or designed for each gene using the *E. coli K12* (NC_000913.3) and *E. coli O157* (NC_002695.1 and NC_002655.2) genomes as references. In silico analysis was performed using SnapGene Viewer version 4.1 [22] and Oligoanalizer 3.1 [23] to evaluate dimers or secondary structures between primers and probes. Primer specificity was evaluated using NBLAST version 2.8.1 [24]. The primer and probe sequences employed are presented in Table 1.

#### 2.2.2. Digital Droplet PCR Validation

A reaction mixture contained 11 μL of 2× ddPCR Supermix for Probes (Bio-Rad 1863024), 800 nM primers, 300 nM probe, and 2 μL of the genomic DNA (gDNA) template (gravimetrically added), with molecular-grade water added to a final volume of 21 μL. A non-template control was included. Both the 21 μL of reaction mix and 70 μL of the droplet generation oil for probes (Bio-Rad, 1863005) were loaded into 8-well cartridges to generate droplets using the QX200 droplet generator (Bio-Rad 1864002). Then, the generated droplets were transferred into a 96-well plate, which was sealed and inserted into a CFX96 touch deep well thermal cycler (Bio-Rad). PCR amplification used the following conditions: 95 °C for 10 min, followed by 40 cycles of 95 °C for 15 s, 58 °C for 60 s, and a final step of 10 °C for 10 min; all steps employed a heating ramp of 0.5 °C/s. The plate was then transferred to a QX200 droplet reader (Bio-Rad, 1864003) to read droplet fluorescence. The intralaboratory ddPCR method validation was performed in simplex mode, and copy number concentration, validation parameters, and decision criteria are described in Table 2. 

#### 2.2.3. Data Analysis

Data acquisition and analysis were performed using Quantasoft software V1.7 (Bio-Rad). Data generated by the QX200 droplet reader were excluded from subsequent analysis when a clog was detected by the Quantasoft software or when a low number of droplets (<10,000) was measured in the PCR mixture. The fluorescence threshold was set manually between the average fluorescence amplitude of positive and negative partitions for each target gene. After exporting, data were analyzed using Microsoft Excel 2016. The following mathematical model (Equation (1)) was used to calculate the sample copy number concentration *C_m_* in copies/μL, using a partition volume of 0.773 ± 0.023 nL (measured in laboratory):

(1) $$C_{m}=\frac{\lambda }{V*d}$$

where *V* is the partition volume, *d* is the total gravimetric dilution, and *λ* represents the copy number per partition, which was calculated from the negative (*N*) and total (*P*) numbers of partitions (droplets) according to Equation (2).

(2) $$\lambda =-Ln\;\left(\frac{N}{P}\right)$$

### 2.3. Genomic DNA Reference Material Production

#### 2.3.1. Culture and DNA Extraction

After *E. coli* O157:H7 (derived from ATCC 35150) cell culture optimization, bacterial cells were harvested from a 200 mL culture via centrifugation at 6000× *g* for 30 min at 4 °C in a Sorvall Lynx 4000 centrifuge. The pellet was washed by centrifugation thrice with 200 mL of 1× phosphate-buffered saline (137 mM NaCl, 2.7 mM KCl, 10 mM Na_2_HPO_4_, 1.8 mM KH_2_PO_4_, pH 7.4). gDNA was extracted using the CTAB/chloroform DNA extraction method [30]. Briefly, the bacterial pellet was resuspended in 7.5 mL of 1× TE buffer and transferred to a 30 mL tube. A total of 7.5 mL of CTAB lysis buffer (8% *w*/*v* CTAB, 40 mM EDTA, 200 mM Tris-HCl, 2.8 M NaCl, and 6% LiCl) and 20 mg/mL proteinase K were added and mixed and then incubated at 65 °C for 1 h. After centrifugation (25,400× *g*, 15 min, 4 °C in Sorvall Lynx 4000 centrifuge using a swinging rotor), the supernatant (the lysate) was transferred to a new 30 mL tube and extracted twice with 1 volume of 24:1 chloroform:isoamyl alcohol. After centrifugation, RNase (20 mg/mL) was added to the aqueous phase and incubated at 37 °C for 1 h with gentle shaking. Then, 10% (*w*/*v*) LiCl was added and incubated for 15 min at 4 °C and extracted with 1 volume of 24:1 chloroform:isoamyl alcohol. The supernatant was precipitated by 1:1.5 isopropyl alcohol and 2.5 M ammonium acetate at −20 °C for 18 h, centrifuged using a swinging rotor at 22,600× *g* for 20 min at 4 °C, washed twice with 70% ethanol, and dried; the DNA pellet was dissolved in 1 mL of 1× TE buffer and stored at −20 °C.

DNA integrity was assessed using 1% agarose gel electrophoresis in 1× TAE buffer (40 mM Tris, 1 mM EDTA, and 20 mM acetic acid, pH 8.5), stained with SYBR^®^ Green DNA dye. DNA purity was assessed using the 260/280 absorbance ratio [31]. The presence of inhibitors was assessed via qPCR amplification, with six gravimetric serial dilutions of gDNA RMs covering four-log concentrations. The acceptability criteria for PCR efficiency were 90–110% [32]. 

#### 2.3.2. Material Preparation

Two 40 mL batches of a DNA stock solution previously measured and quantified using a validated ddPCR method, with a nominal copy number concentration of 100,000 copies/µL and 100 copies/µL (high and low level, respectively), were prepared using gravimetric serial dilutions with TE 1× pH 8.0 as diluent and total yeast RNA as stabilizer (40 ng/µL) [30]. DNA solutions were homogenized in an orbital shaker for 2 h at room temperature. For each batch, a 500 µL DNA solution was dispensed into 1.2 mL labeled polypropylene cryovials (*n* = 80) stored at 4 °C, −20 °C, and −80 °C based on the intended use: homogeneity and stability study, characterization, value assignment and controls, respectively.

#### 2.3.3. Homogeneity Study

ddPCR assays in simplex mode with the *Z3276* assay gene were used to assess the homogeneity of gDNA RM under repeatability conditions: for each batch, eight units were selected, following a systematic random sampling scheme, three replicates for the high level and six replicates for the low level were measured for every unit. The contribution to the combined uncertainty due to the homogeneity (*u_hom_*) was estimated from one-way analysis of variance (ANOVA), according to Equation (3), with vials as the variation factor.

(3) $$u_{bb}=s_{bb}=\sqrt{\frac{{MS}_{between}-{MS}_{within}}{n}}$$

where *u_bb_* is the uncertainty between bottles and is equal to *s_bb_*, the standard deviation between bottles, calculated from the mean square between bottles (*MS_between_*) and the mean squared within bottles *(MS_within_*) and the number of replicates per bottle (*n*).

Since the minimum sample volume (5–20 µL) was smaller than the RM unit volume (500 µL), significant within-unit heterogeneity was checked using six test portions from a unique bottle for each batch via ANOVA, using subsample as the variation factor.

#### 2.3.4. Stability Study

To evaluate the material stability during transport and storage conditions, short- and long-term stability studies were performed at 4 °C and −20 °C, using −80 °C as a reference temperature. For the short-term stability study, following an isochronous design [33], eight units from each batch were stored at 4 °C and −20 °C. At 2, 4, 8, and 12 weeks, two units from each temperature were transferred to the reference temperature (−80 °C) to be measured in triplicate under repeatability conditions by ddPCR using the *Z3276* gene at week 12. The long-term stability study extended the short-term stability study: four different previously selected samples were stored at 4 °C and −20 °C. Following a classical design, at weeks 24 and 60, two units from each temperature were removed and measured, with a control sample (stored at −80 °C), via ddPCR in triplicate using *Z3276*; the data were normalized using the control sample at each time point. Regression analysis was performed in both cases to establish the instability degree, and their contribution to material uncertainty, $u_{lts}$, was calculated from the slope and its standard deviation according to Equation (4):

(4) $$u_{lts}=s(b)(tm)$$

The *u_lts_*, based in the predicted change, is a function of the study time (*tm* = 60 weeks) and the standard error for the estimated slope *s(b).*

#### 2.3.5. Material Characterization and Value Assignment 

To establish the reference value, nine units were randomly selected: three units were measured in triplicate using *Z3276* and *rfbE* assays by ddPCR in simplex mode on three different days. The mean value for each gene was determined, and a consensus value ${(y}_{caract}$) was established for each batch as the mean of both assays. The value assignment for each batch ${(X}_{MR}$) was made following the model described in Equation (5) and is defined for the characterization of the material ${(y}_{caract}$), the homogeneity ($\delta_{hom}$), and stability ${(\delta }_{stb}$):

(5) $$X_{MR}=y_{caract}+\delta_{hom}+\delta_{stb}$$

where $\delta_{hom}$ represent the errors associated with homogeneity and stability, which are usually zero, but not their uncertainties. The material uncertainty was calculated from the combination of the characterization, the homogeneity, and the stability uncertainties (Equation (6)) [34].

(6) $$u\left(X_{MR}\right)=\sqrt{u_{charact}^{2}+u_{hom}^{2}+u_{stb}^{2}}$$

## 3. Results

### 3.1. ddPCR Method Validation

Calibration parameters were evaluated once primer and probe specificities were checked by electrophoresis and melting curve analysis (Supplementary Figure S1).

#### 3.1.1. Selectivity

Eight different *E. coli* serovars of STEC and non-STEC groups as well as closely related and unrelated species were evaluated via qPCR for the presence of all seven target genes (Table 3). The *uidA* gene was amplified from *E. coli* and *Shigella* strains. The *lacY* gene was amplified from *E. coli* and several enterobacteria species. The *eaeA, stx1*, and *stx2* genes were amplified in several STEC strains, whereas the *rfbE* and *Z3276* genes were only amplified in *E. coli O157:H7*. The results show the amplification selectivity of each gene.

#### 3.1.2. Working Interval 

Experimental data fitted a linear regression model in the range of 1.5 to 7900 copies/µL for the seven genes (Table 4; Supplementary Figure S2); however, not all genes met the precision criteria for RSD in the lowest concentration level. Thus, the working range for all genes by the ddPCR method was established as 6.6–7900 copies/µL.

#### 3.1.3. Precision

As the RSD of repeatability was usually higher than the run-to-run variation for ddPCR, only the first one was calculated as method precision. ANOVA was performed for each DNA target gene at each concentration level to calculate the relative repeatability $S_{repeat,rel}$ (Equation (7)).

(7) $$S_{repeat,rel}=\frac{\sqrt{{MS}_{within\;run}}}{C_{sample,\;mean}}$$

where ${MS}_{within\;run}$ is the within-run mean squares and $C_{sample,\;mean}$ is the average copy number concentration calculated over all runs. 

**Figure 1:**
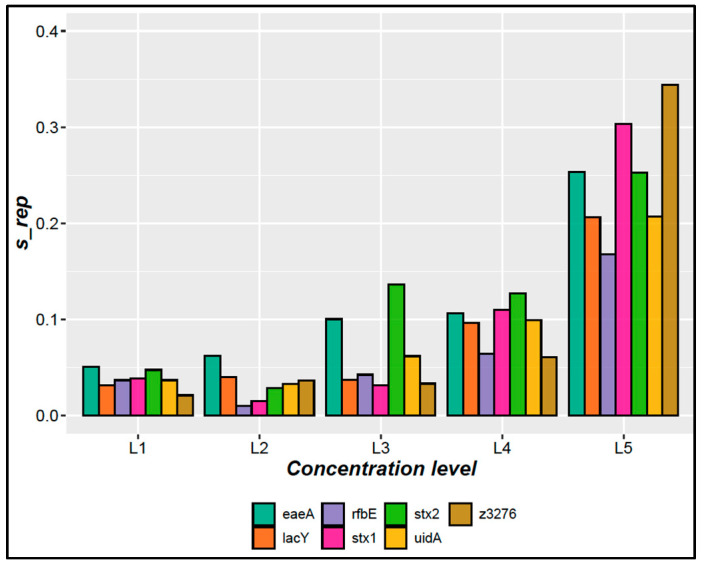
Precision results for the target genes evaluated in this study. Bars represent the relative standard deviation by repeatability for each gene at 5 levels from 1.3 to 7900 copies/µL per reaction. L1 (7920 copies/µL), L2 (718 copies/µL), L3 (66 copies/µL), L4 (6.6 copies/µL), and L5 (1.34 copies/µL).

From the precision data (Supplementary Tables S1–S7), the RSD of the repeatability was calculated for the seven DNA target sequences evaluated over the five concentration levels of the working interval (Figure 1). For L1 to L4 concentration levels, the RSD of the intermediate precision was <13.6% (*stx2*, level 3).

#### 3.1.4. LOQ and LOD

**Figure 2:**
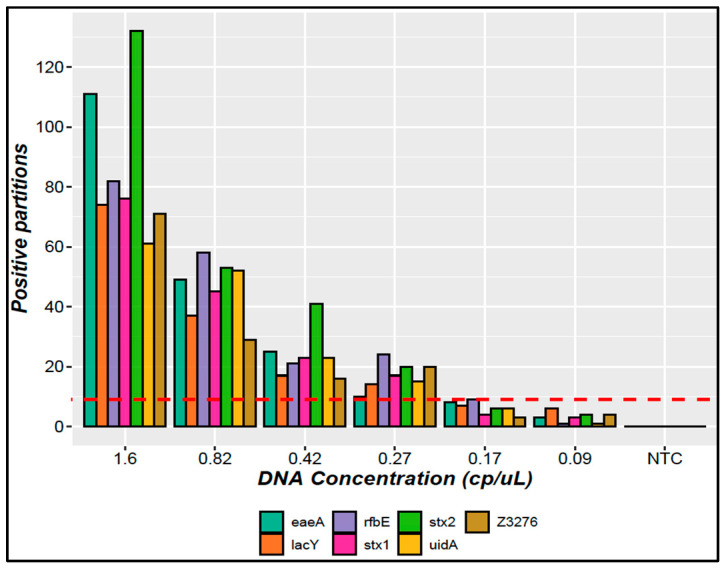
LOD evaluation for the *E. coli* O157:H7 ddPCR method. Bars represent the total positive partitions obtained in all replicates for each gene; the red line represents threshold value: nine positive partitions to define LOD.

The LOQ was established at 6.6 copies/µL based on the working interval and precision results (Figure 1). The LOD was determined as the lowest concentration where at least nine positive partitions in all replicates were detected, with at least ten thousand total partitions per replicate [27]. Six concentration levels under the LOQ were evaluated from 1.60 to 0.08 copies/µL. Figure 2 shows the positive partition distribution related to the concentration level.

Apart from *rfbE*, with 0.17 copies/µL, the other study genes met the acceptance criteria at 0.27 copies/µL in the reaction; thus, this value was established as the LOD for the *E. coli* O157:H7 ddPCR method.

#### 3.1.5. Method Uncertainty

**Figure 3:**
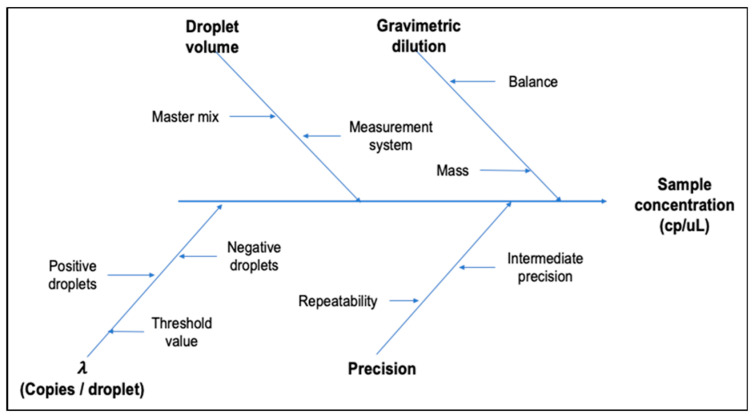
Schematic representation of the principal factors affecting the measurement value and its uncertainty.

The combined standard uncertainty (*u*) for each gene target and concentration level was calculated based on the uncertainty sources identified for the *E. coli* O157:H7 ddPCR method (Figure 3 and Equation (8)).

(8) $$u_{concentration}=\sqrt{u_{Model}^{2}+u_{Precision}^{2}}$$

The mathematical model uncertainty component, based on Equations (1) and (2), is described by Equation (9):

(9) $$u_{Model}=C_{sample}*\sqrt{{\left(\frac{u_{\lambda }}{\lambda }\right)}^{2}+{\left(\frac{u_{d}}{d}\right)}^{2}+{\left(\frac{u_{V}}{V}\right)}^{2}}$$

The precision uncertainty was calculated as follows (Equation (10)), where n is days of measurement.

(10) $$u_{Precision}=\sqrt{\frac{s_{repeat,rel}^{2}}{n}}$$

Table 5 shows the relative combined uncertainty calculated for each DNA target sequence and for each concentration level for the *E. coli* ddPCR method.

### 3.2. Preparation and Quality Control of E. coli DNA Reference 

#### 3.2.1. Culture and DNA Extraction

A total of 1 mL gDNA was obtained from a 0.730 g bacterial pellet, with a concentration of 3728.2 ± 13.7 ng/µL measured by spectrophotometry. The OD260/OD280 and OD260/OD230 ratios of 2.07 and 2.13, respectively, indicate high-quality DNA.

The amplification efficiencies of the different PCR assays calculated via real-time PCR were 98.5%, 97.7%, 99.3%, 99.3%, 108.8%, 97.8%, and 101.4% based on the slope of standard curves *for uidA*, *lacY*, *eaeA*, *rfbE*, *stx1*, *stx2*, and *Z3276* genes, respectively; therefore, no DNA inhibitors were present in the solution. Agarose gel electrophoresis demonstrated that the extracted gDNA had structural integrity. Finally, the estimated concentration of the extracted DNA measured by ddPCR was 1.02 × 10^8^ copies/µL. 

#### 3.2.2. Homogeneity Study

**Figure 4:**
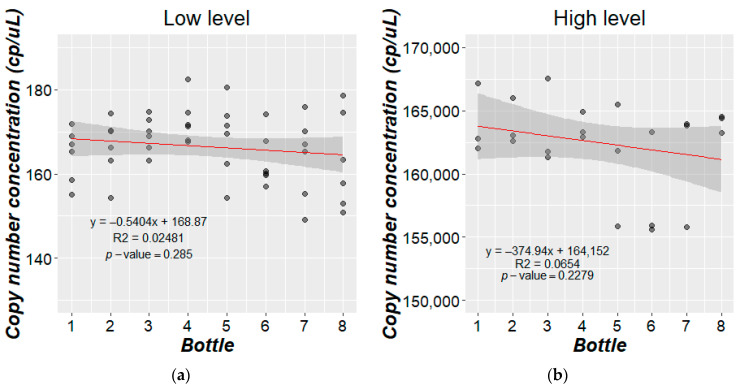
Study of homogeneity between bottles. Packaging trend evaluation for (**a**) low level and (**b**) high level.

Once the high- and low-copy-number-concentration batches were prepared, a processing run effect was checked. For this, the means of the copy number concentrations were plotted against bottle number in preparation order; no significant trends were detected based on regression analysis (Figure 4). Additionally, no significant difference was detected within the heterogeneity for either study materials from the within-unit homogeneity study. The between-unit homogeneity contribution calculated via Equation (3) from ANOVA (Supplementary Table S8) was 1.83 and 806 copies/µL (11% and 0.5%, respectively) for the low- and high-copy-number-concentration levels, respectively.

#### 3.2.3. Stability Study 

**Figure 5:**
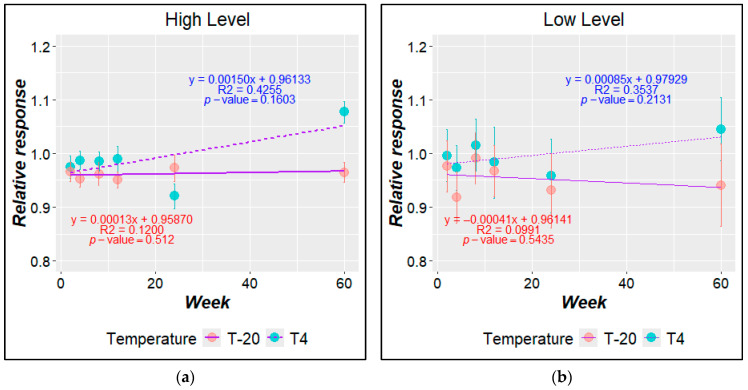
Long-term stability study. Relative response as a function of time for the (**a**) high level and (**b**) low level. Relative response to sample stored at reference temperature (−70 °C).

Based on the regression analysis (and Supplementary Figure S3 and Supplementary Table S9) no significant instability was detected for either the low- or high-concentration-level batches at 4 °C or −20 °C in the short-term stability study. Similarly, the long-term stability study did not exhibit any significant trend in the material concentration during the 60 weeks evaluated at 4 °C or −20 °C (Figure 5).

Considering the dispersion of results around the slope of regression analysis over the evaluated time, the contribution to the uncertainty due to the long-term stability $\left(u_{lts}\right)$ was calculated from the regression data (Supplementary information Table S10). Each batch at each temperature is presented in Table 6.

### 3.3. Material Characterization and Value Assignment

**Figure 6:**
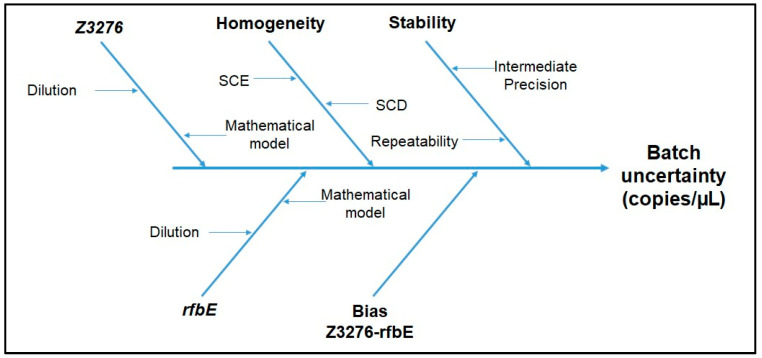
Schematic representation of the main sources of uncertainty in batches.

Figure 6 shows the Ishikawa diagram with the identified uncertainty sources that contribute to the material uncertainty for each batch; *Z3276*, *rfbE*, and Bias *Z3276*-*rfbE* were grouped in the characterization term of Equation (5). Supplementary Figure S4 details the equations used for uncertainty characterization estimation. 

The *Z3276* and *rfbE* values and their uncertainties are derived from the experimental results obtained for the nine units evaluated for three days combined with the uncertainty from the mathematical model (Equation (1)).

**Figure 7:**
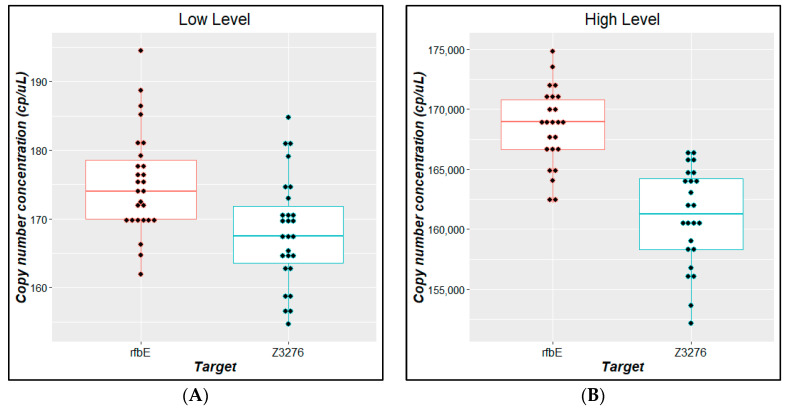
Comparative results for estimated copy number concentration for low-level (**A**) and high-level (**B**) batches with *rfbE* and *Z3276* gene targets.

Figure 7 shows the box plots for *rfbE* and *Z3276* quantification results obtained, for low and high concentration level batches. A *t*-test for independent samples showed a significative difference between them (Supplementary Information SI 1); therefore, the bias between *Z3276* and *rfbE* mean values was included as an uncertainty component (Figure 6), taking the bias as the interval size and assuming a rectangular distribution. The bias uncertainty is derived from the standard deviation from a rectangular distribution between the *Z3276* and the *rfbE* values.

Table 7 presents a summary of the reference values for the batches under study with their associated uncertainty (Supplementary Tables S11–S14 show raw data).

**Figure 8:**
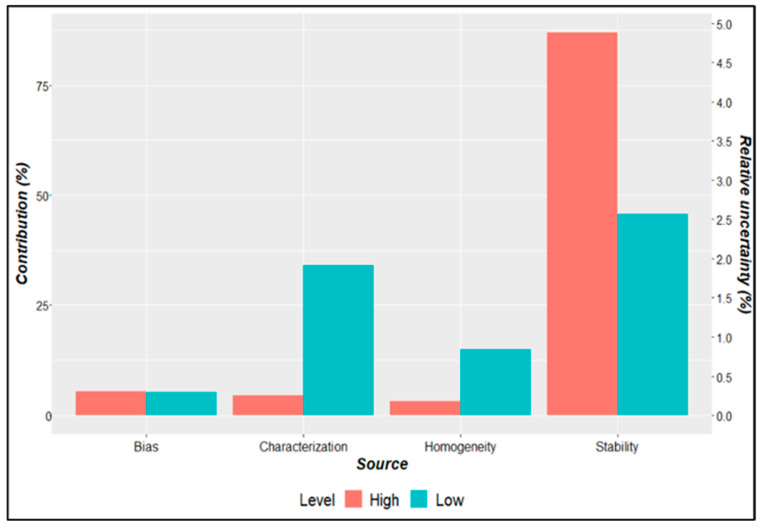
Comparative results for estimated copy number concentration for low-level (blue) and high-level (red) batches with *rfbE* and *Z3276* gene targets.

Figure 8 represents the contribution of each source to the uncertainty, over the mean value and over the combined standard uncertainty in the value assignment process.

Figure 8 demonstrates the bias between the *rfbE* and *Z3276* quantification results and homogeneity was not significant for the standard uncertainty of either material. However, stability appears to be the most important component; and for the low-level concentration, the characterization contributes a further approximately 25% uncertainty.

## 4. Discussion

### 4.1. Method Validation

In this study, we validated a ddPCR method to detect, serotype, and quantify *E. coli* O157:H7 using seven target genes. DNA primers were derived from the reference genomes of *E. coli* O157 (NC_002695.1 and NC_002655.2) and non-pathogenic *E. coli* K12 (NC_000913.3) to provide a suitable method for the identification of different *E. coli* serovars, especially O157:H7, under the same experimental conditions (i.e., primer and probe concentrations, amplification cycles). Although the tests were performed in simplex mode, the selected probes allow the development of duplex PCR assays for improving and optimizing current assays in specialized laboratories. 

As indicated by the selectivity analysis (Table 3), and as expected, the *uidA* gene was amplified from both *E. coli* and *Shigella* strains, although these species can be differentiated based on the amplification of the lactose permease gene *lacY*, which is negative in *Shigella*. Although *Citrobacter freundii* and *Klebsiella pneumonia* lack *lacY*, several reports showed that these species harbor sequences like those of *E. coli lacY*; thus, *lacY* would be useful as a molecular marker only when used in combination with other genes [9]. The *eaeA* gene should theoretically be amplified in all STEC; however, *E. coli O104:H4* tested negative, which may be associated with the multiple variants of this in several STEC serotypes. Thus, degenerate primers should be used for *E. coli* serotype identification [7]. However, *eaeA* was positively amplified in the O157:H7 serovar. The *rfbE* gene was amplified from *E. coli* O157:H7, demonstrating its selectivity toward the STEC O157 group; however, this gene allows discrimination of serotype O157:H7 only in conjunction with *stx1* and *stx2* (which are present in STEC strains) and *Z3276*, which is specific to *E. coli* O157:H7.

Although this amplification method behaves linearly between 1.6 and 7900 copies/µL (in PCR master mix) for all target genes based on linear regression analysis (Table 4; Supplementary Figure S2), the working interval started at 6.6 copies/µL (LOQ) because of the precision criteria (Figure 1). Only *rfbE* showed a precision <20% for all evaluated concentrations, whereas *uidA* and *lacY* had a precision just above the threshold. For *eaeA, stx1, stx2*, and *Z3276*, the precision was >25%. *rfbE* and *Z3276* exhibited an improved precision for the four higher concentrations, indicating that they are the target genes with the highest repeatability. This is important because these genes allow the detection of *E. coli O157* and O157:H7 serovars, and this finding implies that the confidence is higher for these target genes than for the other genes. However, *eaeA* and *stx2* exhibited a larger variability for all concentrations tested. 

For the LOD, nine positive partitions allowed good differentiation of a low-concentration positive sample from the blank, and this is suitable for the intended use of the method. The detection limit was determined as 0.27 gene copies/µL in the reaction mix, which was equivalent to approximately 3 copies/reaction (Figure 2).

Method bias was not evaluated because commercially available quantitative RMs are not available. The trueness of the method was evaluated using a certified identity DNA sample (IRMM 449). For each dilution, the concentration in copies/µL was estimated based on the data of the gravimetric dilutions where the working range could be determined by comparing the gravimetrically determined and measured concentrations. The most important factor contributing to uncertainty was derived from the mathematical model; λ was the predominant source of uncertainty (Table 5). To decrease the uncertainty, some wells could be merged (at least two replicates) in the Quantasoft software to increase the partition number, especially when working with low concentrations near the LOQ.

Based on the results of the validation, this method could quantify the gDNA concentration of *E. coli* serotypes from 6.6 to 7900 copies/µL in the reaction, with a detection limit of 0.27 copies/µL. The method had a precision of 1% to 13.6%, with relative uncertainties between 3.5% and 14.6% in the working interval (Table 5). Due to the selectivity exhibited by the evaluated genes, the method can be used to estimate the DNA concentration in samples during production of RMs of this microorganism and to distinguish between various serotypes of *E. coli* (Table 3).

Considering the uncertainty and precision results (Figure 1; Table 5), the amplification of the *Z3276* and *rfbE* genes was proposed for the characterization of the gDNA of *E. coli* O157:H7 as a candidate for RM, whereas the other genes can provide additional information. Finally, to obtain concentration data at the lowest uncertainties, it is recommended to prepare the reaction mixture close to 1000 copies/µL, where the lowest uncertainty and highest precision were apparent. 

Key experimental information related to ddPCR method can be found in Supplementary Table S15 (dMIQE checklist).

### 4.2. Reference Material Production

Although the extraction process evaluated allowed us to obtain a large amount of DNA, the preparation of these batches was excessive, so the culture can be reduced to 50 mL to start the extraction process to optimize reactions. Although the DNA extraction results showed several higher values for spectrophotometric analysis, indicating the presence of RNA, the qPCR and electrophoresis analysis (to evaluate inhibitor presence and DNA integrity) demonstrated that the method is adequate for semi-preparative extraction of DNA. The small amounts of RNA have no effect on the RM to be prepared since several dilution steps are required to reach the target concentration. Furthermore, during the RM preparation, yeast RNA was added as a stabilizer [30]. Previous studies established that extraction of DNA from solid supports and even solubilization of lyophilized DNA contribute to quantitation variability [35]. Here, we sought to minimize such sources of variability, and the RM candidate of *E. coli* O157:H7 gDNA was prepared as DNA solutions directly suitable for qPCR and ddPCR assays.

Both the high and low RMs prepared here were aqueous solutions of DNA, where the high concentration level can also be considered as diluted and lacked any significative differences among the different subsamples taken for study materials; therefore, the within homogeneity can be considered as zero (Figure 4). Thus, as expected, the maximum degree of heterogeneity found in both RMs was low compared with the assigned value (low level: 1.1% and high level: 0.5%) (Supplementary Table S8). These values can be established as the target homogeneity uncertainty in normalized RM production.

For the short-term stability studies, the temperature used for storage did not significantly affect the concentration value (for the evaluated frame of time) at −20 °C and especially at 4 °C (Supplementary Figure S3 and Table S9). This is crucial if the objective is to use or consume the material rapidly (e.g., in a proficiency test item in an intercomparison study) as this can be transported and kept under refrigeration conditions (4 °C), facilitating the logistics for shipping and preservation of the material.

A similar behavior was observed for both materials at −20 °C and 4 °C for the long-term stability study. Although a significant trend was not observed, the slope and the correlation coefficients based on the regression analysis were higher while the *p*-values were lower for samples stored at 4 °C (Supplementary Table S9). Apparently, this trend was a consequence of high dispersion of results (at week 15) more than the instability degree of the material (Figure 5). Therefore, an intermediate stability point will be required. 

For material characterization, seven validated genes could be used to quantify the RMs. However, using a lower number of units, we chose the two most specific genes that exhibited the best performance in the validation study and measured three different units across three different days to complete nine units once we checked the material was homogeneous and stable. 

Many laboratories perform qPCR assays and use a logarithmic transformation of concentration (base10) to relate the concentration to Ct values and the expected changes associated with an uncertainty of 10% (as a nominal value) in the high- and low-concentration range. This would produce a change in the instrumental response of approximately 1% (Table 8), which could be considered as fit for purpose in measurements by qPCR.

For the uncertainty components, the contributions were similar for both low- and high-copy-number-concentration batches, with stability as the higher contributor to combined standard uncertainty at 60% and 75%, respectively. Thus, to improve stability and material uncertainty, it is necessary to change cryovials to avoid evaporation and changes in the assigned value.

The material developed in this work offers several advantages over others on the market. As gDNA, this contains several target genes used to detect not only *E. coli* O157:H7 but also other serotypes of this genus, which could contribute to the comparability of the results obtained. Once the material is certified, it could be used to quantify microorganisms in samples using calibration curves and to validate various methods that detect serotypes of *E. coli.* Finally, this RM could be used as a quality control in testing laboratories that detect *E. coli* O157:H7 and other strains of this species as well as being used to evaluate the measurement performance of laboratories that use qPCR methods.

## 5. Conclusions

We developed a simplex ddPCR method using seven targets for the identification and quantification of *E. coli* O157:H7. The method, when used with the target gene panel proposed here, can distinguish several serotypes of *E. coli* and quantify *E. coli* serotypes from 6.6 to 7900 copies/µL, with an LOD of 0.27 copies/µL in the PCR mixture; the RSD by repeatability precision was between 1% and 13.6%, and the relative uncertainty was between 3.5% to 14.6% over the working interval. This method can be used to characterize *E. coli* gDNA RMs to estimate sample DNA concentrations and distinguish serotypes. The method validated here could ensure the traceability and comparability of results obtained by PCR methods for the detection and quantification of *E. coli* serotypes in food.

Furthermore, this is the first report that describes the production of quantitative RMs of *E. coli* O157:H7 gDNA for PCR assays and provides guidance to produce similar RMs for the quality control of biologicals. In summary, we produced two batches of a gDNA candidate RM, each of which had 80 bottles. The RM had adequate homogeneity and stability for the intended use. This presentation allows application without high manipulation by the user. Such reference material would improve interlaboratory comparisons and contribute toward standardization of methodologies. The validated assay and the candidate RM produced could contribute to improving food safety and to increasing competitiveness in agricultural product markets.

## Figures and Tables

**Table 1:** Primers and probes of target genes used for *E. coli* ddPCR study.

Microorganism	Gene	Sequences	Fragment Size (bp)	Reference
*Escherichia coli* *Shigella* spp.	*uidA*	GCAAGGTGCACGGGAATATT	75	[8]
		CAGGTGATCGGACGCGT		
		[HEX] CGC CAC TGG CGG AAG CAA CG [BHQ1]		
*Escherichia coli*	*lacY*	TGCTGGCTGGCACTATTATG	153	Adapted from ([9,10])
		GCACTTCAAACTGGCTGGTA		
		[FAM] CCG TTC CTG CTG GTG GGC TGC TT [BHQ1]		
*Diarrheogenic Escherichia coli*	*eaeA*	CATTGATCAGGATTTTTCTGGTGATA	102	[7]
		CTCATGCGGAAATAGCCGTTA		
		[FAM] ATA GTC TCG CCA GTA TTC GCC ACC AAT ACC [BHQ1]		
*Enteropathogenic Escherichia coli* O157 (EPEC)	*rfbE*	TTTCACACTTATTGGATGGTCTCAA	88	[6]
		CGATGAGTTTATCTGCAAGGTGAT		
		[HEX] AGG ACC GCA GAG GAA AGA GAG GAA TTA AGG [BHQ1]		
*Escherichia coli producing Shiga toxins (*STEC*)*	*stx1*	TTTGTTACTGTGACAGCTGAAGCTTTACG	131	[6]
		CCCCAGTTCAATGTAAGATCAACATC		
		[FAM] CTG GAT GAT CTC AGT GGG CGT TCT TAT GTA A [BHQ1]		
	*stx2*	TTTGTCACTGTCACAGCAGAAGCCTTACG	128	
		CCCCAGTTCAGAGTGAGGTCCACGTC		
		[HEX] TCG TCA GGC ACT GTC TGA AAC TGC TCC [BHQ1]		
*Escherichia coli* O157:H7	*Z3276*	CGGGGGATACATTTACGCTT	115	[25,26]
		TTTCTGAGCTGGAACAAGGC		
		[FAM] ACGGTGTTTTCAGGCTTACAGGTCGT [BHQ1]		

FAM: Fluorescein fluorophore, HEX: Hexachlorofluorescein fluorophore, BHQ1: Black hole quencher 1.

**Table 2:** Validation parameters evaluated in ddPCR method validation and acceptance criteria.

Parameter	Description	Criteria
Selectivity	Assessed by qPCR, amplifying each gene against a series of related and unrelated bacterial DNA samples.	Positive amplification in *E. coli* strains. Negative amplification in non-*E. coli* strains.
Working interval	Serial gravimetric dilutions of IRMM 449 over a 5-log DNA concentration range were measured in triplicate for each target gene. Regression analysis was performed to define the working interval.	Correlation coefficient >0.99, a slope significantly (*p* < 0.05) different from zero, an intercept significantly (*p* < 0.05) equal to zero, and a precision <25% as relative standard deviation (RSD).
Precision	Five concentration levels (L): L1 (7920 copies/µL), L2 (718 copies/µL), L3 (66 copies/µL), L4 (6.6 copies/µL), and L5 (1.34 copies/µL) were measured in triplicate on three different days for each target gene.	A repeatability RSD <25% was used as acceptance criterion [27].
Limit of quantification (LOQ)	Defined as the lowest level of the working interval fulfilling linearity and precision criteria.	
Limit of detection (LOD)	Six concentration levels below the quantification limit were evaluated in triplicate.	The LOD was established as the lowest copy number concentration level (copies/μL) where three replicates amplify with at least nine positive partitions together [27].
Uncertainty ^1^	Evaluated for each DNA target in each copy number concentration level, from mathematical model and precision data, according to GUM and EURACHEM guide [28].	

^1^ Measurement uncertainty is not a performance characteristic of a particular measurement procedure but a property of the results obtained using that measurement procedure [29], it was estimated from the validation data.

**Table 3:** Gene amplification profiles indicating *E. coli* O157:H7 ddPCR selectivity.

Group	Species	Reference	*uidA*	*lacY*	*eaeA*	*rfbE*	*Stx1*	*Stx2*	*Z3276*
Gram (+)	*Staphylococcus aureus*	ATCC^®^ 6538	−	−	−	−	−	−	−
	*Staphylococcus aureus*	ATCC^®^ 25923	−	−	−	−	−	−	−
	*Bacillus cereus*	ATCC^®^ 10876	−	−	−	−	−	−	−
	*Enterococcus faecalis*	ATCC^®^ 14506	−	−	−	−	−	−	−
Gram (−)	*Proteus mirabilis*	ATCC^®^ 12453	−	−	−	−	−	−	−
	*Vibrio parahaemolyticus*	ATCC^®^ 17802	−	−	−	−	−	−	−
	*Enterobacter aerogenes*	ATCC^®^ 13048	−	−	−	−	−	−	−
	*Proteus vulgaris*	ATCC^®^ 33420	−	−	−	−	−	−	−
	*Yersinia enterocolitica*	ATCC^®^ 23715	−	−	−	−	−	−	−
*Shigella*	*Shigella boydii*	ATCC^®^ 9207	**+**	−	−	−	−	−	−
	*Shigella sonnei*	ATCC^®^ 9290	**+**	−	−	−	−	−	−
*Escherichia coli*	*Escherichia coli*	ATCC^®^ 25922	**+**	**+**	−	−	−	−	−
	*Escherichia coli*	Donated ^1^	**+**	**+**	−	−	−	−	−
	*Escherichia coli*	NCTC 10538	**+**	**+**	−	−	−	−	−
	*Escherichia coli*	ATCC^®^ 8739	**+**	**+**	−	−	−	−	−
STEC *	*Escherichia coli* O104:H4	ATCC^®^ BAA-2326™	**+**	**+**	−	−	−	**+**	−
	*Escherichia coli* O145:NM	CDC 99-3311	**+**	**+**	**+**	−	**+**	**+**	−
	*Escherichia coli* O157:H7	ATCC^®^ 700728™	**+**	**+**	**+**	**+**	−	−	**+**
	*Escherichia coli* O157:H7	ATCC^®^ 35150™	**+**	**+**	**+**	**+**	**+**	**+**	**+**
*Salmonella*	*Salmonella Thyphimurium*	Donated ^2^	−	−	−	−	−	−	−
	*Salmonella Thyphi*	Donated ^2^	−	−	−	−	−	−	−
	*Salmonella enteritidis*	Donated ^2^	−	−	−	−	−	−	−

^1^ Donated by the Medicine Laboratory of National University of Colombia, ^2^ Salmonella DNA, donated by the National Institute of Health (INS), Microbiology Group. * STEC, Shiga-Toxin-Producing *Escherichia coli.*

**Table 4:** Linear regression analysis for *E. coli O157:H7* quantification by ddPCR.

Gene	Slope	Intercept	Correlation Coefficient (r^2^)
*uidA*	1,516,070 ± 7553	−5.3 ± 13.9	0.99980
*lacY*	1,652,697 ± 14,605	−17.8 ± 26.5	0.99938
*eaeA*	1,732,747 ± 15,360	−21.2 ± 28.8	0.99937
*rfbE*	1,688,518 ± 19,028	−12.1 ± 34.8	0.99899
*stx1*	1,683,566 ± 19,537	−23.5 ± 35.4	0.99892
*stx2*	1,894,405 ± 11,534	−22.3 ± 27.3	0.99970
*Z3276*	1,612,203 ± 12,551	−6.5 ± 22.9	0.99952

**Table 5:** Relative standard uncertainty for each gene by the *E. coli* ddPCR method.

Concentration Level (Copies/µL)	*uidA*	*lacY*	*eaeA*	*rfbE*	*stx1*	*stx2*	*Z3276*
7920	3.5%	4.1%	8.4%	4.1%	4.1%	4.6%	3.4%
718	2.7%	3.0%	4.2%	2.5%	2.0%	2.2%	2.9%
66	4.8%	4.2%	6.6%	5.0%	4.2%	8.0%	4.4%
6.6	13.2%	12.9%	12.3%	7.4%	13.4%	13.4%	11.3%

**Table 6:** Stability uncertainty for candidate RM batches.

Batch	Storage Temperature			
	4 °C	−20 °C	4 °C	−20 °C
	u_stb_	u_rel_ (%)	u_stb_	u_rel_ (%)
Low level	5.95	3.4%	6.37	3.7%
High level	8632	5.2%	1822	1.1%

u_stb_: Stability uncertainty, u_rel_: Relative uncertainty.

**Table 7:** Assigned value and uncertainty for evaluated batches.

**Batch**	**Gene *Z3276***		**Gene *rfbE***		**Uncertainty Sources**			
	**Value** **(Copies/µL)**	**u*_Z3276_***	**Value**	**u*_rfbE_***	**u_bias_**	**u_charac._**	**u_homog._**	**u_stab._**
High level	161,117	3975	168,610	3858	2158	1945	824	8627
Low level	168	8	175	8	2	5	2	6
**Value Assignment**								
	**Batch**	**Value**	**u**	**Relative u (%)**	**k**	**U**	**Relative U (%)**	
	High level	164,770	9140	5.5	2	18,280	11.1	
	Low level	172	8	4.9	2	17	9.8	

U = expanded uncertainty, u = combined standard uncertainty.

**Table 8:** Variation in instrumental response (Ct) as a function of 10% uncertainty in the assigned value.

Level	Concentration (Copies/µL)	log^10^ Concentration	Interpolated Ct *	∆ Ct	∆ Ct Rel (%)
High	100,000	5.00	23.4	0.29	1.24
	90,000	4.95	23.5		
	110,000	5.04	23.3		
Low	100	2	33.4	0.29	0.87
	90	1.95	33.5		
	110	2.04	33.2		

* Estimated from concentration assuming a 100% efficiency reaction (slope −3.32 and intercept of 40).

## Data Availability

The original contributions presented in the study are included in the article and Supplementary Material, further inquiries can be directed to the corresponding author.

## Supplementary Materials

The following supporting information can be downloaded at: https://www.mdpi.com/article/10.3390/mps7060094/s1; Figure S1. Electrophoresis of amplification products of *E. coli* O157:H7 gene targets; Figure S2. Linear interval for DNA targets in *E. coli* O157:H7 gene quantification by ddPCR; Tables S1–S7. Data of precision experiments performed on three different days at five sample concentration levels with three replicates for each sequence target; Table S8. Analysis of variance of homogeneity between bottles of pilot batches;Table S9. Regression analysis results for short- and long-term stability study for high-concentration-level (HL) and low-concentration-level (LL) materials at 4 °C and −20 °C; Table S10. Uncertainty of short- and long-term stability for high-concentration-level (HL) and low-concentration-level (LL) materials at 4 °C and −20 °C; Supplementary Information SI 1; Table S11. Characterization results of batch of 100,000 copies/μL with *Z3276*; Table S12. Characterization results of batch of 100,000 copies/μL with *rfbE*; Table S13. Characterization results of batch of 100 copies/μL with *Z3276*; Table S14. Characterization results of batch of 100 copies/μL with *rfbE*; Figure S3. Short-term stability study results (relative to controls) for high- and low-copy-number-concentration batches. Figure S4. Uncertainty estimation candidate for reference material; Table S15. dMIQE checklist.

## Abbreviations

$C_{sample,mean}$: Average copy number concentration	Ct: Cycle threshold
$C_{sample}$: Sample concentration	CTAB: Hexadecyltrimethylammonium bromide
${MS}_{between}$: Mean square between bottles	d: Gravimetric dilution
${MS}_{within}$: Mean squared within bottles	ddPCR: Droplet digital PCR
${MS}_{witinrun}$: Within-run mean squares	DNA: Deoxyribonucleic acid
$S_{repeat,rel}$: Relative repeatability	dPCR: Digital PCR
$X_{MR}$: Value assignment	EDTA: Ethylenediaminetetraacetic acid
$s_{bb}$: Between-bottle standard deviation	gDNA: Genomic DNA
$u_{hom}$: Homogeneity uncertainty	LOD: Limit of detection
$u_{Model}$: Model uncertainty	LOQ: Limit of quantification
$u_{Precision}$: Precision uncertainty	N: Negative partitions
$u_{V}$: Volume uncertainty	P: Total partitions
$u_{bb}$: Between-bottle uncertainty	PCR: Polymerase chain reaction
$u_{charact}$: Uncertainty of material characterization	qPCR: Real-time PCR
$u_{concentration}$: Concentration uncertainty	RM Reference material
$u_{d}$: Dilution uncertainty	RNA: Ribonucleic acid
$u_{lts}$ Long-term stability	RNase: Ribonuclease
$u_{stb}$: Stability uncertainty	RSD: Relative standard deviation
$u_{\lambda }$: Lambda uncertainty	STEC: Shiga-toxin-producing *E. coli*
$y_{caract}$: Characterization of the material	TE: Tris-EDTA
$\delta_{hom}$: Error/bias due to homogeneity	u: Standard uncertainty
$\delta_{stb}$: Error/bias due to stability	V: Partition volume
ANOVA: Analysis of variance	λ: Copy number per partition
ATCC: American Type Culture Collection	s(b): Standard deviation between bottles
cdPCR: Chamber digital PCR	tm: Monitoring time
C_m_: Copy number concentration in copies/μL	$u\left(X_{MR}\right)$: Value assignment uncertainty
