# Supplementary material for: Digital PCR Validation for Characterization of Quantitative Reference Material of Escherichia coli O157:H7 Genomic DNA
Source: Methods Protoc. 2024 Nov 15;7(6):94. doi: 10.3390/mps7060094 (PMC11587158; doi:10.3390/mps7060094)
Supplement: Supplementary file 1 [file mps-07-00094-s001.zip › mps-3067632-supplementary.pdf]

Supplementary information for article “Digital PCR validation for characterization of quantitative reference material of Escherichia coli O157:H7 genomic DNA”

## Method validation

**Figure S1**. E*lectrophoresis of amplification products of E. coli O157:H7 gene targets.*

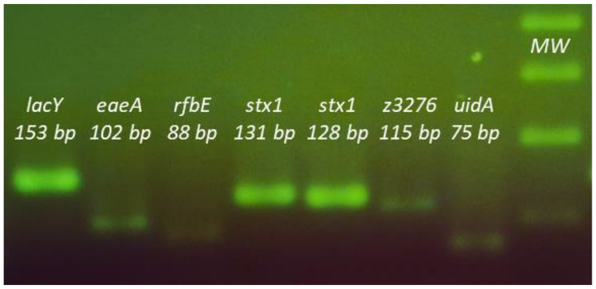


2% agarose electrophoresis (80V for 90 min) Lane 1 to 7: Amplicon *lacY*, *eaeA*, *rfbE*, *stx1*, *stx2*, *Z3276 and uidA* respectively, Lane 8: EZ Load 100 bp Molecular Ruler. Negative controls for each gene did not amplify (not shown).

**Figure S2**. *Linear interval for DNA targets in E. coli O157:H7 gene quantification by ddPCR*

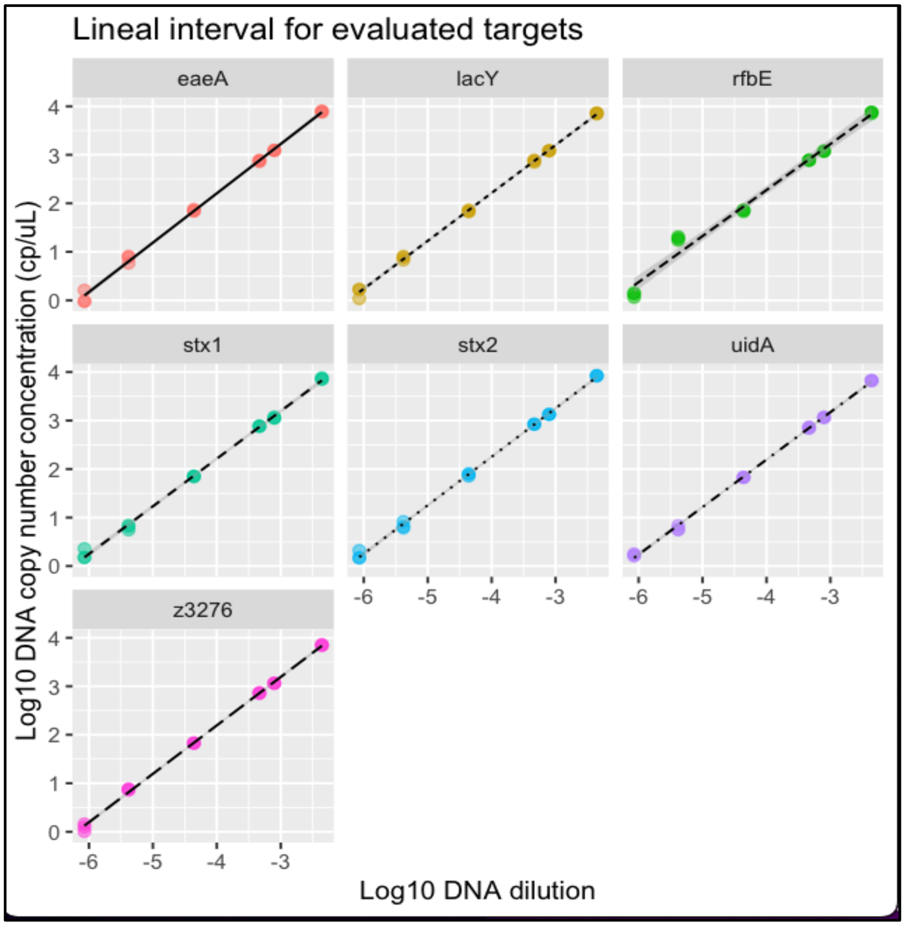


Amplification curves and linearity graphs of the amplification cycle depending on the logarithm of the MRC IRMM 449 DNA dilution by qPCR.

**Tables S1-S7.** Data of precision experiments performed in three different days at five sample concentration levels with three replicas for each sequence target.

**Table S1**. *uidA* measurement results in ddPCR reaction

| Level | Day | Replicate 1 | Replicate 2 | Replicate 3 | Dilution |
|---|---|---|---|---|---|
| 1 | 1 | 6540.13 | 6697.62 | 6794.56 | 8.66E-02 |
| | 2 | 6794.82 | 7533.69 | 7062.06 | 9.28E-02 |
| | 3 | 6530.17 | 6545.06 | 6896.68 | 8.92E-02 |
| 2 | 1 | 683.96 | 712.77 | 740.21 | 9.12E-02 |
| | 2 | 639.56 | 660.39 | 614.79 | 7.83E-02 |
| | 3 | 681.27 | 652.63 | 672.99 | 8.24E-02 |
| 3 | 1 | 67.10 | 68.38 | 66.68 | 8.75E-02 |
| | 2 | 59.70 | 68.85 | 56.71 | 8.08E-02 |
| | 3 | 61.40 | 62.90 | 59.34 | 8.10E-02 |
| 4 | 1 | 5.75 | 5.53 | 6.85 | 8.47E-02 |
| | 2 | 6.85 | 7.16 | 6.16 | 8.03E-02 |
| | 3 | 5.06 | 6.12 | 5.13 | 8.04E-02 |
| 5 | 1 | 1.69 | 1.60 | 1.80 | 8.55E-02 |
| | 2 | 1.68 | 1.33 | 1.33 | 8.35E-02 |
| | 3 | 1.05 | 2.02 | 1.48 | 8.03E-02 |

**Table S2.** *lacY* measurement results in ddPCR reaction.

| Level | Day | Replicate 1 | Replicate 2 | Replicate 3 | Dilution |
|---|---|---|---|---|---|
| 1 | 1 | 6898.69 | 7222.34 | 7401.78 | 8.54E-02 |
| | 2 | 7678.82 | 8255.54 | 7688.78 | 9.30E-02 |
| | 3 | 8031.36 | 7980.63 | 8355.61 | 9.10E-02 |
| 2 | 1 | 696.06 | 779.84 | 759.19 | 9.12E-02 |
| | 2 | 691.99 | 672.64 | 662.83 | 7.83E-02 |
| | 3 | 725.92 | 685.97 | 687.31 | 8.24E-02 |
| 3 | 1 | 67.25 | 67.67 | 73.66 | 8.75E-02 |
| | 2 | 63.28 | 66.85 | 65.71 | 8.08E-02 |
| | 3 | 61.33 | 60.74 | 63.80 | 8.10E-02 |
| 4 | 1 | 6.73 | 8.12 | 7.71 | 8.47E-02 |
| | 2 | 7.28 | 5.77 | 6.43 | 8.03E-02 |
| | 3 | 5.99 | 5.29 | 5.67 | 8.04E-02 |
| 5 | 1 | 1.69 | 1.67 | 1.08 | 8.55E-02 |
| | 2 | 1.17 | 0.99 | 1.72 | 8.35E-02 |
| | 3 | 1.48 | 1.59 | 1.48 | 8.03E-02 |

**Table S3**. *eaeA* measurement results in ddPCR reaction

| Level | Day | Replicate 1 | Replicate 2 | Replicate 3 | Dilution |
|---|---|---|---|---|---|
| 1 | 1 | 7536.62 | 7786.61 | 8102.18 | 8.87E-02 |
| | 2 | 8969.36 | 8174.37 | 8417.44 | 9.47E-02 |
| | 3 | 9724.67 | 9159.13 | 10300.70 | 8.57E-02 |
| 2 | 1 | 753.44 | 780.90 | 720.52 | 8.97E-02 |
| | 2 | 719.06 | 714.51 | 595.67 | 8.04E-02 |
| | 3 | 741.42 | 739.41 | 749.84 | 8.08E-02 |
| 3 | 1 | 68.36 | 75.74 | 70.42 | 8.54E-02 |
| | 2 | 67.24 | 57.72 | 66.29 | 7.85E-02 |
| | 3 | 71.56 | 71.60 | 55.37 | 7.72E-02 |
| 4 | 1 | 7.73 | 5.81 | 8.16 | 8.45E-02 |
| | 2 | 6.60 | 6.54 | 6.79 | 7.96E-02 |
| | 3 | 6.96 | 6.27 | 6.75 | 7.85E-02 |
| 5 | 1 | 0.96 | 0.95 | 1.63 | 8.52E-02 |
| | 2 | 1.06 | 1.25 | 0.82 | 8.40E-02 |
| | 3 | 1.07 | 1.59 | 1.20 | 7.93E-02 |

**Table S4**. *rfbE* measurement results in ddPCR reaction

| Level | Day | Replicate 1 | Replicate 2 | Replicate 3 | Dilution |
|---|---|---|---|---|---|
| 1 | 1 | 7025.54 | 7612.30 | 7633.26 | 8.64E-02 |
| | 2 | 7859.07 | 8008.88 | 7753.30 | 9.16E-02 |
| | 3 | 8026.80 | 8103.37 | 7509.71 | 9.11E-02 |
| 2 | 1 | 769.02 | 766.27 | 787.97 | 9.02E-02 |
| | 2 | 715.78 | 705.07 | 716.13 | 7.87E-02 |
| | 3 | 694.99 | 692.05 | 699.02 | 7.92E-02 |
| 3 | 1 | 67.04 | 71.84 | 73.73 | 8.60E-02 |
| | 2 | 63.97 | 64.42 | 68.45 | 7.71E-02 |
| | 3 | 66.54 | 63.37 | 65.73 | 8.14E-02 |
| 4 | 1 | 20.54 | 17.23 | 18.89 | 8.48E-02 |
| | 2 | 18.76 | 17.07 | 18.36 | 7.92E-02 |
| | 3 | 18.82 | 18.20 | 17.02 | 7.90E-02 |
| 5 | 1 | 1.33 | 1.44 | 1.15 | 8.43E-02 |
| | 2 | 1.41 | 1.55 | 1.24 | 8.47E-02 |
| | 3 | 0.96 | 1.40 | 0.85 | 8.08E-02 |

**Table S5**. stx1 measurement results in ddPCR reaction

| Level | Day | Replicate 1 | Replicate 2 | Replicate 3 | Dilution |
|---|---|---|---|---|---|
| 1 | 1 | 7005.08 | 7281.22 | 7673.33 | 8.56E-02 |
| | 2 | 7648.85 | 7814.37 | 7899.21 | 9.24E-02 |
| | 3 | 7569.11 | 6978.06 | 7019.57 | 8.77E-02 |
| 2 | 1 | 758.91 | 749.29 | 781.04 | 9.02E-02 |
| | 2 | 695.38 | 698.07 | 686.75 | 7.87E-02 |
| | 3 | 699.22 | 683.37 | 700.67 | 7.92E-02 |
| 3 | 1 | 70.58 | 72.01 | 69.02 | 8.60E-02 |
| | 2 | 63.97 | 64.42 | 68.45 | 7.71E-02 |
| | 3 | 67.98 | 67.26 | 63.87 | 8.14E-02 |
| 4 | 1 | 6.98 | 5.53 | 6.46 | 8.48E-02 |
| | 2 | 7.11 | 6.39 | 6.07 | 7.92E-02 |
| | 3 | 6.56 | 5.13 | 5.43 | 7.90E-02 |
| 5 | 1 | 1.48 | 1.52 | 2.30 | 8.43E-02 |
| | 2 | 0.74 | 1.23 | 1.77 | 8.47E-02 |
| | 3 | 1.84 | 1.48 | 1.08 | 8.08E-02 |

**Table S6**. stx2 measurement results in ddPCR reaction

| Level | Day | Replicate 1 | Replicate 2 | Replicate 3 | Dilution |
|---|---|---|---|---|---|
| 1 | 1 | 8553.49 | 8414.37 | 8198.42 | 8.71E-02 |
| | 2 | 11086.21 | 9778.56 | | 9.14E-02 |
| | 3 | 11193.89 | 11182.05 | | 8.76E-02 |
| 2 | 1 | 832.77 | 853.86 | 835.48 | 8.79E-02 |
| | 2 | 775.25 | 771.97 | 749.30 | 7.91E-02 |
| | 3 | 805.62 | 775.25 | 736.90 | 8.05E-02 |
| 3 | 1 | 71.06 | 80.93 | 77.32 | 8.43E-02 |
| | 2 | 72.67 | 68.24 | 71.31 | 7.93E-02 |
| | 3 | 80.04 | 67.43 | 49.44 | 7.81E-02 |
| 4 | 1 | 6.50 | 6.06 | 8.28 | 8.49E-02 |
| | 2 | 6.25 | 7.15 | 6.72 | 7.68E-02 |
| | 3 | 6.73 | 8.26 | 6.59 | 7.76E-02 |
| 5 | 1 | 2.10 | 1.50 | 1.45 | 8.52E-02 |
| | 2 | 0.93 | 1.45 | 1.08 | 8.54E-02 |
| | 3 | 1.60 | 0.91 | 1.03 | 7.86E-02 |

**Table S7**. Z3276 measurement results in ddPCR reaction

| Level | Day | Replicate 1 | Replicate 2 | Replicate 3 | Dilution |
|---|---|---|---|---|---|
| 1 | 1 | 6873.09 | 7322.71 | 7001.59 | 8.62E-02 |
| | 2 | 7188.09 | 7373.52 | 7241.81 | 8.66E-02 |
| | 3 | 7419.37 | 7572.51 | 7556.88 | 9.28E-02 |
| 2 | 1 | 702.38 | 732.91 | 740.48 | 8.87E-02 |
| | 2 | 689.01 | 653.75 | 615.59 | 8.04E-02 |
| | 3 | 686.06 | 671.08 | 693.41 | 7.96E-02 |
| 3 | 1 | 67.64 | 66.15 | 67.71 | 8.64E-02 |
| | 2 | 64.09 | 63.35 | 58.65 | 7.85E-02 |
| | 3 | 60.03 | 61.54 | 63.66 | 7.84E-02 |
| 4 | 1 | 7.56 | 7.63 | 7.18 | 8.69E-02 |
| | 2 | 6.06 | 6.04 | 6.91 | 7.96E-02 |
| | 3 | 6.11 | 6.81 | 6.89 | 7.96E-02 |
| 5 | 1 | 1.45 | 1.25 | 1.01 | 8.55E-02 |
| | 2 | 0.94 | 1.36 | 0.65 | 8.40E-02 |
| | 3 | 1.00 | 0.82 | 1.78 | 7.70E-02 |

# Reference material production

**Table S8**. Analysis of variance of homogeneity between bottles of pilot batches

| Origin of variations | Sum of squares | Degrees of freedom | Average of squares | $F_{cal}$ | $p_{(0,05)}$ | $F_{crit}$ |
|---|---|---|---|---|---|---|
| **Low level concentration batch100 copies/µL** | | | | | | |
| **Between groups** | 501 | 7 | 71.6 | 1.16 | 0.34 | 2.25 |
| **Within groups** | 2465 | 40 | 61.6 | | | |
| **Total** | 2966 | 47 | | | | |
| **High level concentration Batch 100.000 copies/µL** | | | | | | |
| **Between groups** | 91,965,676 | 7 | 13,137,953 | 1.17 | 0.37 | 2.66 |
| **Within groups** | 179,023,138 | 16 | 11,188,946 | | | |
| **Total** | 270,988,815 | 23 | | | | |

**Figure S3**

Short term stability study results (relative to controls) for high- and low-level copy number concentration batches.

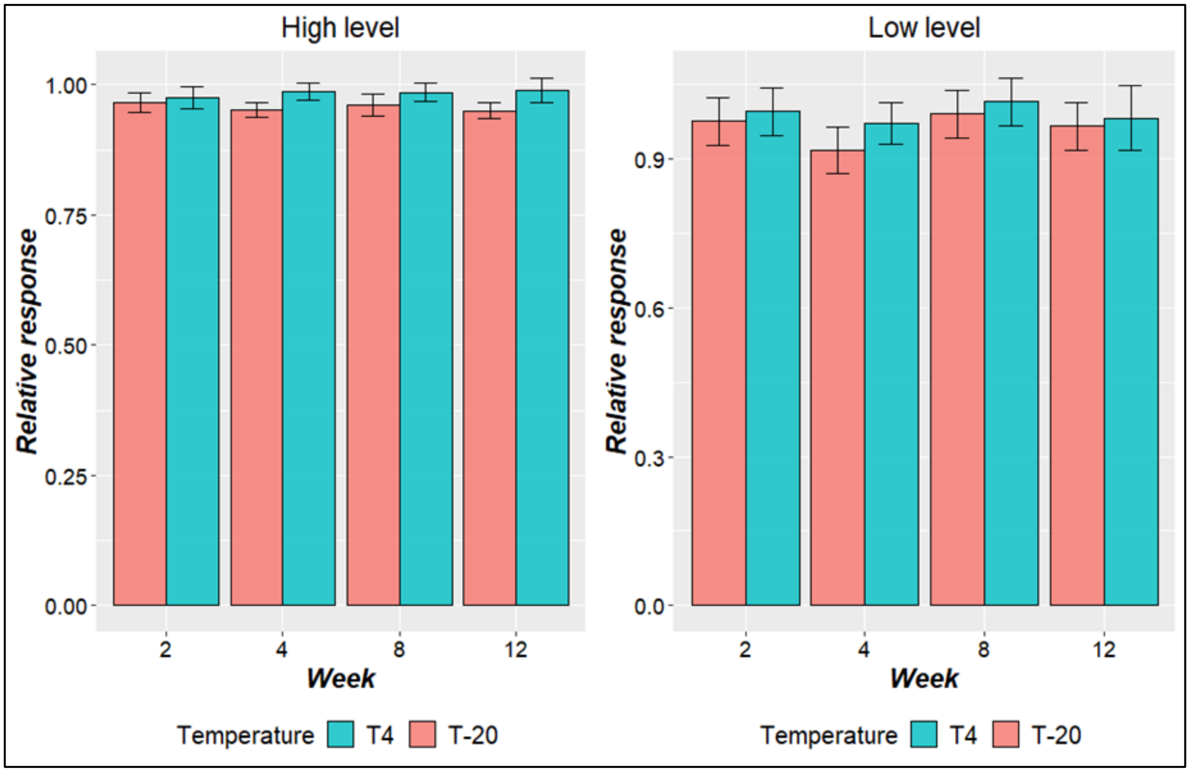


**Table S9.** Regression analysis results for short- and long-term stability study for high (HL) and low (LL) concentration level materials at 4ºC and -20ºC.

| Study | Material/condition | Slope | Intercept | R2 | p-value |
|---|---|---|---|---|---|
| STS | LL-4ºC | 0.000244 | 0.9902 | 0.003526 | 0.9406 |
| | LL -20ºC | 0.00200 | 0.9499 | 0.07817 | 0.7204 |
| | HL-4ºC | 0.00108 | 0.9768 | 0.5628 | 0.2498 |
| | HL -20ºC | -0.00096 | 0.9637 | 0.3217 | 0.4328 |
| LTS | LL-4ºC | 0.00085 | 0.9792 | 0.3537 | 0.2131 |
| | LL -20ºC | -0.00041 | 0.9614 | 0.09906 | 0.5435 |
| | HL-4ºC | 0.00150 | 0.9613 | 0.4255 | 0.1603 |
| | HL -20ºC | 0.00013 | 0.9587 | 0.1200 | 0.5012 |

**Table S10.** Uncertainty of short- and long-term stability for high (HL) and low (LL) concentration level materials at 4ºC and -20ºC.

| Condition | Slope | Standard error | relative uncertainty | u absolut uncertainty |
|---|---|---|---|---|
| LTS 4 HL | 0.00150 | 0.0008727 | 0.052362 | 8632 |
| LTS-20 HL | 0.00013 | 0.0001843 | 0.011058 | 1823 |
| LTS 4 LL | 0.00085 | 0.000577 | 0.03462 | 5.95 |
| LTS-20 LL | -0.00041 | 0.0006171 | 0.037026 | 6.37 |

## Supplementary Information SI 1

T.test (performed with R Version 4.4.3)

Low concentration level
Two Sample t-test
data: LL_Z3276 and LL_rfbE
t = -3.3751, df = 52, p-value = 0.001402
alternative hypothesis: true difference in means is not equal to 0
95 percent confidence interval:
-11.218848 -2.852748
sample estimates:
mean of x mean of y
168.1533 175.1891

High Concentration level
Two Sample t-test
data: HL_Z3276 and HL_rfbE
t = -7.0148, df = 46, p-value = 8.658e-09
alternative hypothesis: true difference in means is not equal to 0
95 percent confidence interval:
-9619.664 -5329.899
sample estimates:
mean of x mean of y
161032.7 168507.5

**Table S11.** Characterization results of batch 100,000 copies/µL with Z3276

| Day | Sample | Result by days | | | | | Total result | |
|---|---|---|---|---|---|---|---|---|
| | | Value | $u_{comb.}$ | $u_{rel}$ | Average | $u_{comb.}$ Day | Average | $u_{comb.}$ |
| 1 | 190401-08P-26 | 133,250 | 2203 | 1.7% | 161,795 | 1806 | 161,117 | 3975 |
| | 190401-08P-42 | 161,762 | 2619 | 1.6% | | | | |
| | 190401-08P-66 | 161,827 | 2486 | 1.5% | | | | |
| 2 | 190401-08P-10 | 162,900 | 2894 | 1.8% | 162,945 | 1775 | | |
| | 190401-08P-50 | 164,668 | 2462 | 1.5% | | | | |
| | 190401-08P-58 | 161,266 | 2288 | 1.4% | | | | |
| 3 | 190401-08P-18 | 154,070 | 2568 | 1.7% | 158,613 | 2777 | | |
| | 190401-08P-34 | 159,595 | 2354 | 1.5% | | | | |
| | 190401-08P-75 | 162,174 | 2415 | 1.5% | | | | |

**Table S12.** Characterization results of batch 100,000 copies/µL with *rfbE*

| Day | Sample | Result by days | | | | | Total result | |
|---|---|---|---|---|---|---|---|---|
| | | Value | u comb. | u rel | Average | u comb. Day | Average | u comb. |
| 1 | 190401-08P-26 | 141,085 | 2166 | 1.5% | 169,427 | 2879 | 168,610 | 3858 |
| | 190401-08P-42 | 171,661 | 2526 | 1.5% | | | | |
| | 190401-08P-66 | 167,194 | 2610 | 1.6% | | | | |
| 2 | 190401-08P-10 | 169,707 | 2522 | 1.5% | 170,103 | 1526 | | |
| | 190401-08P-50 | 171,153 | 2450 | 1.4% | | | | |
| | 190401-08P-58 | 169,449 | 2463 | 1.5% | | | | |
| 3 | 190401-08P-18 | 164,628 | 2404 | 1.5% | 166,299 | 1702 | | |
| | 190401-08P-34 | 159,595 | 2354 | 1.5% | | | | |
| | 190401-08P-75 | 162,174 | 2415 | 1.5% | | | | |

**Table S13.** Characterization results of batch 100 copies/µL with Z3276

| Day | Sample | Result by days | | | | | Total result | |
|---|---|---|---|---|---|---|---|---|
| | | Value | u comb. | u rel | Average | u comb. Day | Average | u comb. |
| 1 | 190401-10P-24 | 160,18 | 6.52 | 4.1% | 164 | 4 | 168 | 8 |
| | 190401-10P-51 | 163,79 | 6.29 | 3.8% | | | | |
| | 190401-10P-59 | 167,05 | 6.29 | 3.8% | | | | |
| 2 | 190401-10P-16 | 170,29 | 6.44 | 3.8% | 169 | 4 | | |
| | 190401-10P-67 | 171,42 | 6.55 | 3.8% | | | | |
| | 190401-10P-76 | 166,29 | 6.25 | 3.8% | | | | |
| 3 | 190401-10P-08 | 167,32 | 6.48 | 3.9% | 171 | 4 | | |
| | 190401-10P-32 | 171,96 | 6.44 | 3.7% | | | | |
| | 190401-10P-40 | 175,08 | 6.41 | 3.7% | | | | |

**Table S14.** Characterization results of batch 100 copies/µL with *rfbE*

| Day | Sample | Result by days | | | | | Total result | |
|---|---|---|---|---|---|---|---|---|
| | | Value | u comb. | u rel | Average | u comb. Day | Average | u comb. |
| 1 | 190401-10P-24 | 177 | 6.44 | 3.6% | 177 | 4 | 175 | 8 |
| | 190401-10P-51 | 181 | 6.74 | 3.7% | | | | |
| | 190401-10P-59 | 175 | 7.81 | 4.5% | | | | |
| 2 | 190401-10P-16 | 183 | 7.24 | 4.0% | 177 | 6 | | |
| | 190401-10P-67 | 170 | 6.32 | 3.7% | | | | |
| | 190401-10P-76 | 179 | 6.63 | 3.7% | | | | |
| 3 | 190401-10P-08 | 171 | 6.82 | 4.0% | 171 | 4 | | |
| | 190401-10P-32 | 171 | 6.46 | 3.8% | | | | |
| | 190401-10P-40 | 172 | 6.67 | 3.9% | | | | |

**Figure S4**. *Uncertainty estimation candidate for reference material*

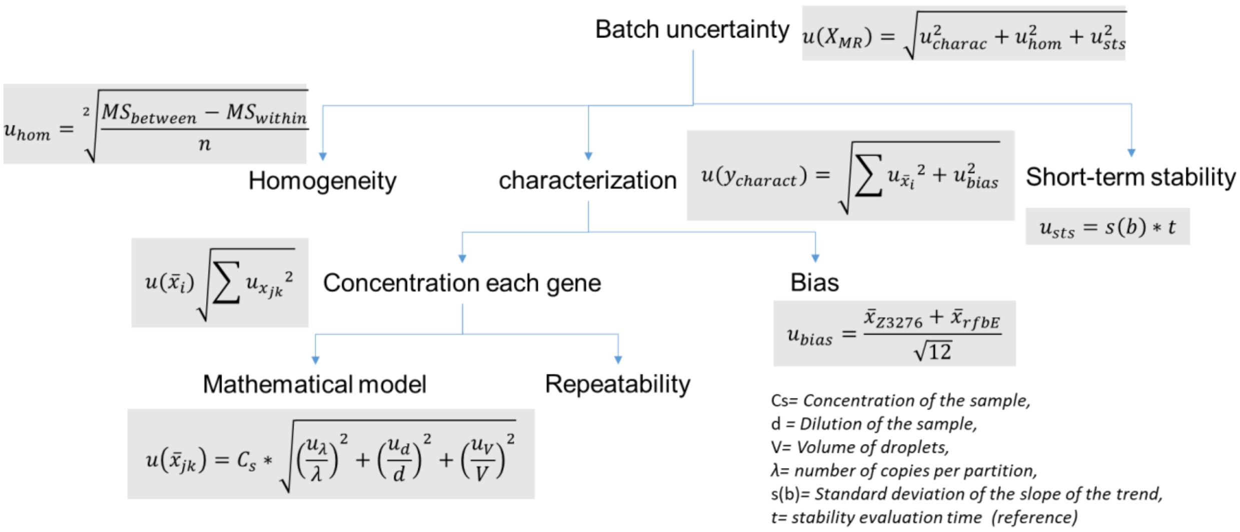


**Table S15**. dMIQE checklist

| ITEM TO CHECK | IMPORTANCE | Comments |
|---|---|---|
| **EXPERIMENTAL DESIGN** | | |
| Definition of experimental and control groups | **E** | Not applicable |
| Number within each group | **E** | Not applicable |
| Assay carried out by core lab or investigator's lab? | D | Investigator's lab |
| Power analysis | D | Not applicable |
| **SAMPLE** | | |
| Description | **E** | Included in manuscript |
| Volume or mass of sample processed | **E** | Included in manuscript |
| Microdissection or macrodissection | **E** | Not applicable |
| Processing procedure | **E** | Not applicable |
| If frozen - how and how quickly? | **E** | Not applicable |
| If fixed - with what, how quickly? | **E** | Not applicable |
| Sample storage conditions and duration (especially for FFPE samples) | **E** | Included in manuscript |
| **NUCLEIC ACID EXTRACTION** | | |
| Quantification - instrument/method | **E** | Included in the manuscript |
| Storage conditions: temperature, concentration, duration, buffer | **E** | Included in the manuscript |
| DNA or RNA quantification | **E** | Included in the manuscript |
| Quality/integrity-instrument/method; e.g. RIN/RQI and trace or 3':5' | **E** | Included in the manuscript |
| Template structural information | **E** | Not applicable |
| Template modification (digestion, sonication, pre-amplification etc.) | **E** | Not applicable |

| ITEM TO CHECK | IMPORTANCE | Comments |
| --- | --- | --- |
| Template treatment (initial heating or chemical denaturation) | **E** | Not applicable |
| Inhibition dilution or spike; | **E** | Not applicable |
| DNA contamination assessment of RNA sample | **E** | Included in the manuscript |
| Details of DNase treatment where performed | **E** | Included in the manuscript |
| Manufacturer of reagents used and catalogue number | D | Not applicable |
| Storage of nucleic acid: temperature, concentration, duration, buffer | **E** | Included in the manuscript |
| **REVERSE TRANSCRIPTION (If necessary)** | | |
| cDNA priming method + concentration | **E** | Not applicable |
| One or two step protocol | **E** | Not applicable |
| Amount of RNA used per reaction | **E** | Not applicable |
| Detailed reaction components and conditions | **E** | Not applicable |
| RT efficiency | D | Not applicable |
| Estimated copies measured with and without addition of RT | D | Not applicable |
| Manufacturer of reagents used and catalogue number | D | Not applicable |
| Reaction volume (for two step reverse transcription reaction) | D | Not applicable |
| Storage of cDNA: temperature, concentration, duration, buffer | D | Not applicable |
| **dPCR TARGET INFORMATION** | | |
| Sequence accession number | **E** | Included in manuscript |
| Location of amplicon | D | uidA:  2369650 - 2396742<br>lacY: 418928 - 419080<br>eaeA: 4667314 - 4667415<br>rfbE: 2852517 - 2852604<br>stx1: 2996304 - 2996434<br>stx2: 1352836 - 1352963<br>Z3276: 2933746 - 2933860<br>Reference location in *Escherichia coli O157:H7 str. EDL933, complete sequence.* GenBank NC_002655 |
| Amplicon length | **E** | Included in manuscript |
| In silico specificity screen (BLAST, etc) | **E** | Included in supplementary information |
| Pseudogenes, retropseudogenes or other homologs? | D | Not applicable |
| Sequence alignment | D | Included in supplementary information |
| Secondary structure analysis of amplicon and GC content | D | Included in supplementary information |
| Location of each primer by exon or intron (if applicable) | **E** | Not applicable |
| Where appropriate, which splice variants are targeted? | **E** | Not applicable |
| **dPCR OLIGONUCLEOTIDES** | | |
| Primer sequences and/or amplicon context sequence | **E** | Included in manuscript |
| RTPrimerDB Identification Number | D | Not included |
| Probe sequences | D | Included in manuscript |

| ITEM TO CHECK | IMPORTANCE | Comments |
|---|---|---|
| Location and identity of any modifications | **E** | Not applicable |
| Manufacturer of oligonucleotides | D | Included in manuscript |
| Purification method | D | RPC for primers and RP HPLC/dual HPLC for probes |
| **dPCR PROTOCOL** | | |
| Complete reaction conditions | **E** | Included in manuscript |
| Reaction volume and amount of RNA/cDNA/DNA | **E** | Included in manuscript |
| Primer, (probe), Mg++ and dNTP concentrations | **E** | Included in manuscript |
| Polymerase identity and concentration | **E** | Included in manuscript |
| Buffer/kit Catalogue No and manufacturer | **E** | Included in manuscript |
| Exact chemical constitution of the buffer | D | Not applicable |
| Additives (SYBR Green I, DMSO, etc.) | **E** | Not applicable |
| Plates/tubes Catalogue No and manufacturer | D | Included in manuscript |
| Complete thermocycling parameters | **E** | Included in manuscript |
| Reaction setup | D | Included in manuscript |
| Gravimetric or volumetric dilutions (manual/robotic) | D | Manual |
| Total PCR reaction volume prepared | D | Included in manuscript |
| Partition number | **E** | 13000 - 19000 |
| Individual partition volume | **E** | Included in manuscript |
| Total volume of the partitions measured (effective reaction size) | **E** | 10.6 – 15.6 uL |
| Partition volume variance/standard deviation | D | Not applicable |
| Comprehensive details and appropriate use of controls | **E** | Included in manuscript |
| Manufacturer of dPCR instrument | **E** | Included in manuscript |
| **dPCR VALIDATION** | | |
| Optimisation data for the assay | D | Not included in manuscript |
| Specificity (when measuring rare mutations, pathogen sequences etc.) | **E** | Included in manuscript |
| Limit of detection of calibration control | D | Included in manuscript |
| If multiplexing, comparison with singleplex assays | **E** | Not included in manuscript |
| **DATA ANALYSIS** | | |
| Average copies per partition (λ or equivalent) | **E** | L1: 7,920 cp/uL<br>L2: 718 cp/uL<br>L3: 66 cp/uL<br>L4: 6.6 cp/uL<br>L5: 1.3 cp/uL |
| dPCR analysis program (source, version) | **E** | Included in manuscript |
| Outlier identification and disposition | **E** | Included in manuscript |
| Results of NTCs | **E** | Included in manuscript |
| Examples of positive(s) and negative experimental results as supplemental data | **E** | Included in supplementary information |
| Where appropriate, justification of number and choice of reference genes | **E** | Included in manuscript |

| ITEM TO CHECK | IMPORTANCE | Comments |
|---|---|---|
| Where appropriate, description of normalization method | **E** | Not applicable |
| Number and concordance of biological replicates | D | Not applicable |
| Number and stage (RT or qPCR) of technical replicates | **E** | Included in manuscript |
| Repeatability (intra-assay variation) | **E** | Included in manuscript |
| Reproducibility (inter-assay/user/lab etc. variation) | D | Included in manuscript |
| Experimental variance or confidence interval | **E** | Included in manuscript |
| Statistical methods used for analysis | **E** | Included in manuscript |
| Data submission using RDML | D | Not included in manuscript |

E: Essential information, D: Desirable information
